# *Agrobacterium rhizogenes*-Mediated Transformation of the Parasitic Plant *Phtheirospermum japonicum*

**DOI:** 10.1371/journal.pone.0025802

**Published:** 2011-10-03

**Authors:** Juliane K. Ishida, Satoko Yoshida, Masaki Ito, Shigetou Namba, Ken Shirasu

**Affiliations:** 1 Graduate School of Agricultural and Life Sciences, University of Tokyo, Tokyo, Japan; 2 Plant Science Center, RIKEN, Yokohama, Japan; 3 Graduate School of Bioagricultural Sciences, Nagoya University, Nagoya, Japan; Deutsches Krebsforschungszentrum, Germany

## Abstract

**Background:**

Plants within the *Orobanchaceae* are an agriculturally important group of parasites that attack economically important crops to obtain water and nutrients from their hosts. Despite their agricultural importance, molecular mechanisms of the parasitism are poorly understood.

**Methodology/Principal Findings:**

We developed transient and stable transformation systems for *Phtheirospermum japonicum*, a facultative parasitic plant in the *Orobanchaceae*. The transformation protocol was established by a combination of sonication and acetosyringone treatments using the hairy-root-inducing bacterium, *Agrobacterium rhizogenes* and young seedlings. Transgenic hairy roots of *P. japonicum* were obtained from cotyledons 2 to 3 weeks after *A. rhizogenes* inoculation. The presence and the expression of transgenes in *P. japonicum* were verified by genomic PCR, Southern blot and RT-PCR methods. Transgenic roots derived from *A. rhizogenes-*mediated transformation were able to develop haustoria on rice and maize roots. Transgenic roots also formed apparently competent haustoria in response to 2,6-dimethoxy-1,4-benzoquinone (DMBQ), a haustorium-inducing chemical. Using this system, we introduced a reporter gene with a Cyclin B1 promoter into *P. japonicum*, and visualized cell division during haustorium formation.

**Conclusions:**

We provide an easy and efficient method for hairy-root transformation of *P. japonicum*. Transgenic marker analysis revealed that cell divisions during haustorium development occur 24 h after DMBQ treatment. The protocols described here will allow functional analysis of genes involved in plant parasitism.

## Introduction

Parasitic plants obtain water and nutrients directly from their host plants through a specialized structure, the haustorium [1]. Among the parasitic genera, *Striga* and *Orobanche* (*Orobanchaceae*) are some of the most destructive agricultural pests in the world [2]. The genus *Striga* infests an estimated 20 to 40 million hectares in sub-Saharan Africa, causing yield losses exceeding 1 billion USD per year, and thus directly affecting the lives of more than 100 million subsistence farmers [2]–[4]. The cultivated area affected by *Orobanche* is estimated to exceed 16 million hectares in the Mediterranean and Near East regions [4].

In nature, parasitic plants initiate haustoria upon the recognition of host roots, and through the action of host-derived chemicals known collectively as Haustorium Inducing Factors (HIF). HIF include the flavonoids xenognosin A and B, and a range of quinones, such as 2,6 dimethoxy-1,4-benzoquinone (DMBQ) [5]–[7]. Haustorium induction by HIF is mediated by the accumulation of reactive oxygen species catalyzed by a quinone oxidoreductase (QR1), which plays a crucial role in *Triphysaria versicolor* (Orobranchaceae) haustorium initiation [8]. During the initial stages of haustorial development, root epidermal cells differentiate and proliferate root hair-like structures while the cortical cells begin to swell. After the pro-haustorium becomes slightly visible, successive cell divisions occur in epidermal and cortical cells, leading to formation of the mature haustorium [9]–[11].

While the morphology of haustorium development has been described, little molecular description of this process has been reported. The discovery of new genes involved in haustorium development will provide insights into how parasitic plants control interactions with their hosts. Moreover, these genes may represent potential targets for engineering genetic resistance to parasitic weeds. Large-scale expressed sequence tag (EST) projects were thus carried out in *Triphysaria* (*T. versicolor* and *T. pusilla*) [12] and *Striga hermonthica* [13]. Currently, *T. versicolor*, together with *S. hermonthica* and *Orobanche* (*Phelipanche*) *aegyptiaca*, is the subject of massive transcriptome sequencing with next generation sequencers (http://ppgp.huck.psu.edu). However, the lack of an easy and efficient genetic transformation protocol for parasitic plants represents a bottleneck for the functional analyses of their genes. Currently, among the parasitic angiosperms, only *T. versicolor* has been successfully transformed [14].

*Phtheirospermum japonicum* is a facultative parasite closely related to the agricultural pests *Striga* and *Orobanche* [15]. The autumn-flowering herb *P. japonicum* is native in East Asia and parasitize a broad range of hosts though it does not have any economic importance. This parasite can be easily cultivated and bred in the laboratory condition with the short life cycle (∼3 months). Therefore *P. japonicum* represents an excellent parasitic model plant. In contrast to *T. versicolor* [8], *P. japonicum* is a self-compatible plant which facilitates genetic experimentation in future. In this manuscript we report a transformation protocol for *P. japonicum* using *Agrobacterium rhizogenes*. Since *P. japonicum* roots produce strong oxidative injuries in response to established methods for *Agrobacterium*-mediated transformation, we employed weak sonication to inoculate the bacteria into plant tissues, and showed that transgenic roots attach to host plants and form haustoria in response to HIF. Using this system, we visualized cell division during early haustorium development with a yellow fluorescence protein (YFP) reporter driven by the CyclinB1 promoter.

## Results and Discussion

### Wound responses may interfere with *A. rhizogenes*–based transformation methods using hypocotyl-dip or needle inoculation

**Figure 1:**
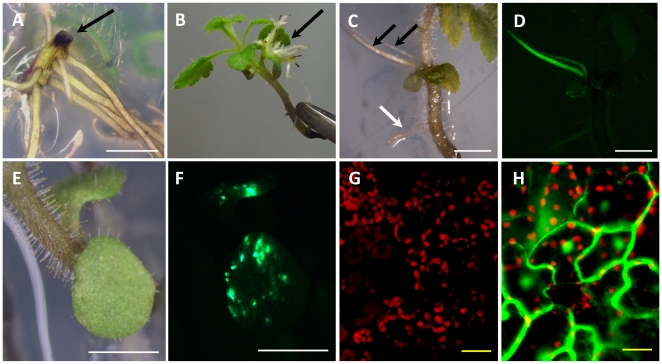
Stable and transient transformation of *P. japonicum* mediated by *A. rhizogenes*. Transformation induced by *A. rhizogenes* ATCC15834. **A**. Five-week-old plants showing accumulation of black substance(s) at the wound site after hypocotyl-cutting infection method. **B**. Transformed roots emerged from cotyledons 5 weeks after *A. rhizogenes* inoculation by the SAAT method. The black arrow indicates transformed roots. **C** and **D** GFP-fluorescing transformed roots observed under bright field (C) or fluorescent (D) microscopy. The black arrows point to fluorescent roots and the white arrow to non-fluorescent root. **E** and **F** Transiently-transformed cotyledons observed under bright field (E) or fluorescent (F) microscopy. **G** and **H** Confocal micrograph of cotyledon leaves. Non-transformed (G) and transiently-transformed (H). Red color corresponds to autofluorescence from chlorophyll. White bars correspond to 2 mm and yellow bars to 20 µm.

To develop an efficient *P. japonicum* transformation method, we first tested protocols established for *Lotus japonicus* [16], *Triphysaria* [14] and *Phaseolus* ssp. [17]. Briefly, hypocotyls of 5 day-old *P. japonicum* seedlings which had had their roots removed were inoculated with a suspension of *A. rhizogenes*, or alternatively the bacteria were injected directly into intact plants with a hypodermic needle. Transformation was evaluated by the detection of green fluorescence protein (GFP) under control of a Cauliflower Mosaic Virus *35S* promoter (CaMV 35S). However, no hairy roots emerged with detectable GFP fluorescence with any of the previously published protocols. Instead, wounded sites accumulated black substance(s), most likely oxidized phenolics [18], [19] (Fig. 1A). A similar reaction was also observed in cut roots without *A. rhizogenes* inoculation, suggesting that this is a typical wounding response in *P japonicum*.

### Sonication assists *A. rhizogenes*-based transformation of P. japonicum

To overcome the wound response interference problem, we used ultrasound sonication to induce minor injuries in plant tissues [20]. Sonication-assisted *Agrobacterium* transformation (SAAT) protocols have been applied with success in several crop plants, such as soybeans, cowpea, wheat and maize [21], [20]. However, as these transformation protocols utilized *A. tumefaciens*, we needed to adapt the method for hairy root transformation using *A. rhizogenes*. Intact three-day-old *P. japonicum* seedlings were sonicated in the *A. rhizogenes* suspension, followed by vacuum treatment and co-incubation. Hairy roots emerged from *P. japonicum* cotyledons 2–3 weeks after the inoculation (Fig 1B). GFP fluorescence was detected in the new emerging roots, indicating that stable hairy root transformation was established (Fig.1C, D). In some cases, GFP fluorescence appeared as multiple spots in a cotyledon (Fig.1E, F), but cotyledons were unable to produce fluorescent roots within 4–5 weeks after inoculation. To verify that the fluorescence observed here is due to GFP expressed in plant cells and not in bacteria, we analyzed the fluorescent cotyledons using the confocal microscopy (Fig. 1G,H), The GFP fluorescence was observed in cytosol and nuclear of cotyledon cells (Fig. 1H), confirming that the method can also result in transient transformation. When stably transformed hairy roots were excised and cultured in hormone-free media, these roots accumulated black substance(s) and subsequently died 3–4 weeks after the treatment. The accumulation of wound response compounds was not observed in unexcised transformed roots which can retain GFP expression at least for 2 months, thus composite plants harboring transgenic hairy roots were maintained intact. Using the *A. rhizogenes*-based method described above, we were able to obtain transgenic roots in 4–5 weeks, which allow the direct analysis of essential genes for root parasitism, skipping time-consuming hormone treatment for root induction required for conventional *A. tumefaciens* transformation protocols.

#### Transgene integration in hairy roots

**Figure 2:**
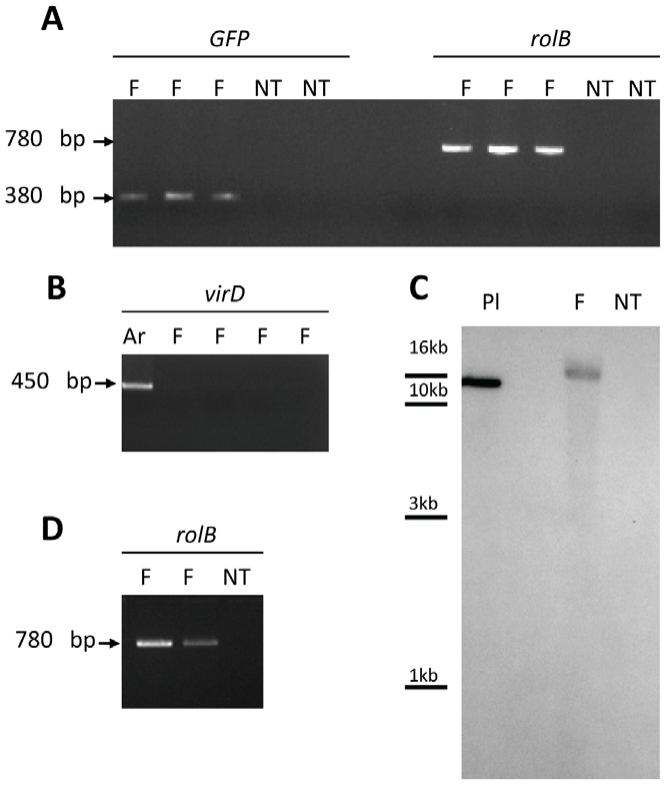
Detection of transgenes by PCR and RT-PCR. **A-B** PCR analysis of genomic DNA isolated from *P. japonicum* fluorescent hairy roots (F) and non-transformed tissues (NT). **A**. Amplification of *GFP* and *rolB* fragments with expected sizes (380 bp and 780 bp, respectively). **B**. No amplification of *virD1* fragment (450 bp) in fluorescent tissues (F), as positive control a diluted ATCC15834 bacterial suspension (Ar) was used. **C**. Southern blot of genomic DNA extracted from fluorescent (F) and non-transformed (NT) *P. japonicum* roots. DNA was digested with *Eco*RI. The positive control (Pl) corresponds to linearised pBCR101 plasmid (30 ng). **D**. RT-PCR analysis of the *rolB* gene using total RNAs extracted from *P. japonicum* fluorescent roots (F) and non-transformed tissues (NT).

The presence and expression of transgenes in the P. japonicum genome were confirmed by genomic PCR, Southern blot, and RT-PCR. PCR amplification confirmed the presence of GFP and the TL region (rolB) in the genome of transgenic roots (Fig. 2A). To determine if the hairy root samples retained contaminating bacteria, PCR was used to detect virD1, a bacterial gene which is not integrated into the plant genome. There was no specific amplification of virD1 in any hairy root samples, but the expected 450 bp virD1 fragment was present in controls (Fig. 2B). Southern blot analysis was also used to confirm T-DNA integration into the P. japonicum genome. Genomic DNA of transformed roots was digested with EcoRI, which does not cut within the T-DNA, and hybridized with a labelled 380 bp GFP fragment. No hybridization signal was observed in non-transformed plants. In contrast, the presence of GFP fragment was detected in fluorescent root, showing that T-DNA containing GFP was integrated into transgenic root genome (Fig. 2C). RT-PCR using RNA extracted from hairy roots and non-transformed tissues was used to confirm that the transgenes were transcribed in planta. RolB gene expression was detected in two independent transgenic lines (Fig. 2D), indicating that the transgenes were stably transformed and expressed.

#### Optimization of A. rhizogenes-meditated transformation

**Figure 3:**
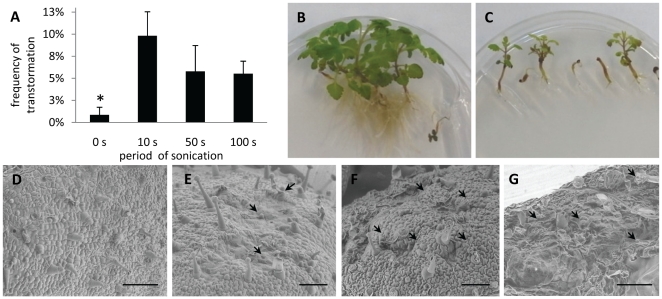
Effect of sonication treatment on *P. japonicum* plants. **A** Transformation efficiency with *A. rhizogenes* strain ATCC15834 and sonication (0, 10, 50 and 100 s) followed by application of vacuum for 5 min. Bars represent the means and standard error of at least 3 independent experiments with 20–40 plants each. Statistical significance is marked by * (P < 0.1). **B** and **C** Photographs of 5-week-old plants without sonication treatment (B) and submitted to 100 s sonication at the age of 3 days old(C). **D** to **G** Scanning electron micrographs of a 3-day-old cotyledons without sonication (D) and cotyledons after 10 s (E), 50 s (F) and 100 s (G) sonication. Arrows point to damage on cotyledon surfaces caused by sonication. Bars correspond to 125 µm

To evaluate the effects of sonication period on transformation efficiency, seedlings were sonicated for up to 100 s. Cotyledons immersed in *A. rhizogenes* ATCC15384 suspension without sonication averaged less than 1% transformation. Stable transformation events increased with sonication from 10 to 100 seconds (Fig. 3A). Although transformation occurred during all of the sonication periods tested, longer exposures resulted in severe damage to plants (Fig. 3 B, C). To assess wounding intensity due to sonication, *P. japonicum* cotyledons were examined with an electron microscope after 0 s, 10 s, 50 s and 100 s sonication. Non-sonicated tissues were apparently healthy (Fig. 3D), but a large number of micro-wounds were observed on the surfaces of sonicated cotyledons (Fig. 3 E–G). Sonication-induced lesions were adequate for efficient transformation with 10 s treatment, so 10 s was adopted as the standard treatment length in all subsequent experiments.

**Figure 4:**
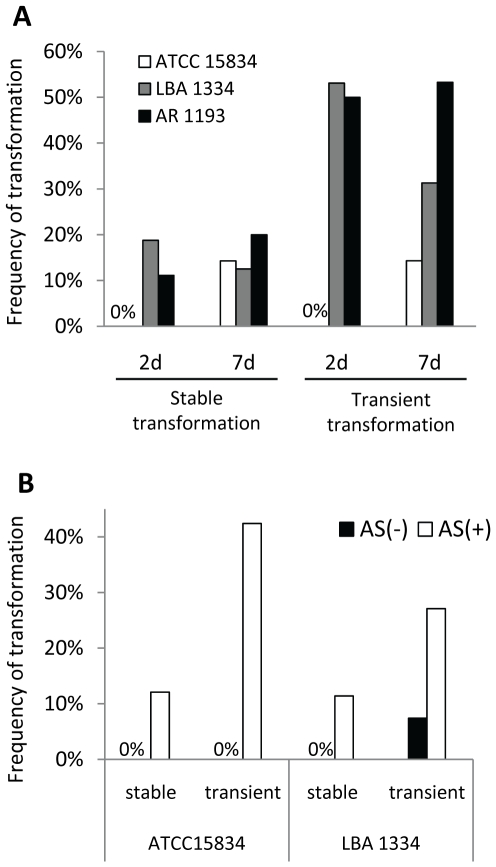
Optimization of factors influencing stable transformation of *P. japonicum*. Frequency of stable transformation in *P. japonicum s*eedlings submitted to different treatments. **A** Transformation efficiency in 3-day-old seedlings infected with strains ATCC15834, LBA1334 and AR1193 and co-cultivated for 2 or 7 days. The data show representative results from one of two independent experiments using 20 to 60 plants each. **B** Effect of acetosyringone (AS) on transformation efficiency. *S*eedlings were co-cultivated with *A. rhizogenes* strains ATCC15834 and LBA1334 in media with or without 100 µM AS for 2 days for LBA1334, and 7 days for ATCC15834. 4–5 weeks after the inoculation the transformation frequency was scored. The data show representative results from one of at least two independent experiments using 20 to 60 plants each.

**Figure 5:**
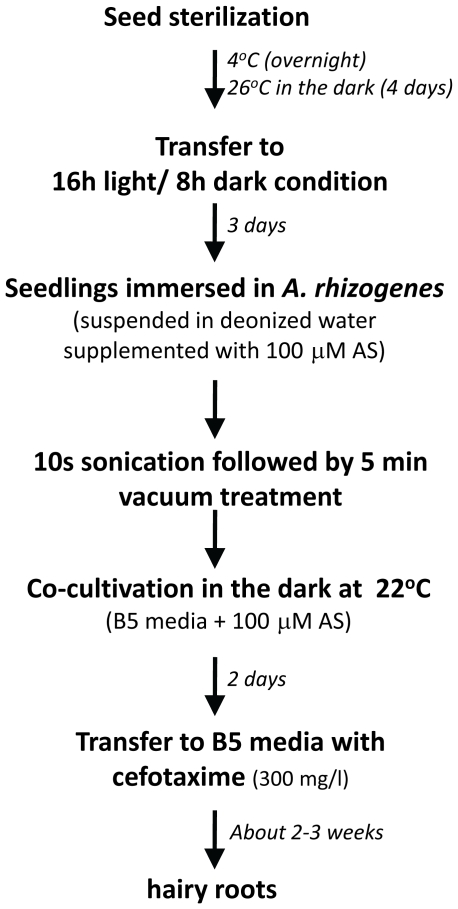
A flowchart for hairy root transformation in *P. japonicum*.

*Agrobacterium*-mediated transformation efficiency often differs according to the bacterial strain used. Therefore *A. rhizogenes* strains ATCC15834 [22], LBA1334 [23] and AR1193 [24] (Table 1), were tested for *P. japonicum* transformation. Due to growth rate variations of the different strains, we also investigated if the duration of co-incubation affects transformation efficiency. The highest transformation rates were obtained using LBA1334 and AR1193 (Fig. 4A); both strains showed similar efficiency in both stable and transient transformation when co-cultivated with *P. japonicum* for 2 or 7 days (Fig. 4A). However, the 2 day protocol is more appropriate, since a longer period allowed overgrowth of *A. rhizogenes* on plants and thus an increase of dead tissues. The bacteria suspension media used in the SAAT methods established in other plant species contain additive compounds such as phytohormone auxin to increase the frequency of gene transfer and induce root formation [25], the surfactant Silwet L-77 and/or acetosyringone, a *vir* gene inducer [26], [27].To evaluate the efficacy of these compounds in transformation, we tested suspension media in combination with Silwet L-77 and/or the auxin 1-naphthaleneacetic acid (NAA). Seedlings immersed in bacterial suspension media had similar transformation rates to those immersed in a water suspension (Table 2). Among the compounds tested, only acetosyringone showed a clear effect on stable transformation. This phenolic compound also greatly increased the percentage of tissues with transient GFP expression (Fig. 4B). These results indicate that the addition of acetosyringone during the infection process significantly enhances stable transformation and transient expression regardless of the bacterial strain. A summary of the optimized transformation protocol is shown in Figure 5.

**Table 1:** *Agrobacterium rhizogenes* strains.

strain	plasmid	construction
ATCC 15834	pBCR101	35S::GFP/GUS
LBA 1334	pBCR101	35S::GFP/GUS
AR 1193	pBCR101	35S::GFP/GUS
AR1193	CYCB1;2 pro::YFP	CYCB1;2 pro::YFP

**Table 2:** Efficiency of stable transformation after the addition of Silwet L-77 and/or NAA into bacterial suspension.

bacterial suspension media	Transformation frequency % (± SD) with each treatment			
	none	Silwet L-77	NAA	Silwet l77 & NAA
MS salt	4 (±0.81)	4 (±0.81)	5 (±0.5)	7 (±2.0)
	N = 67	N = 65	N = 62	N = 64
water	6 (±1)	6 (±1)	6 (±2)	4 (±0.57)
	N = 46	N = 63	N = 46	N = 48

#### Transgenic roots connect to the host via haustoria

**Figure 6:**
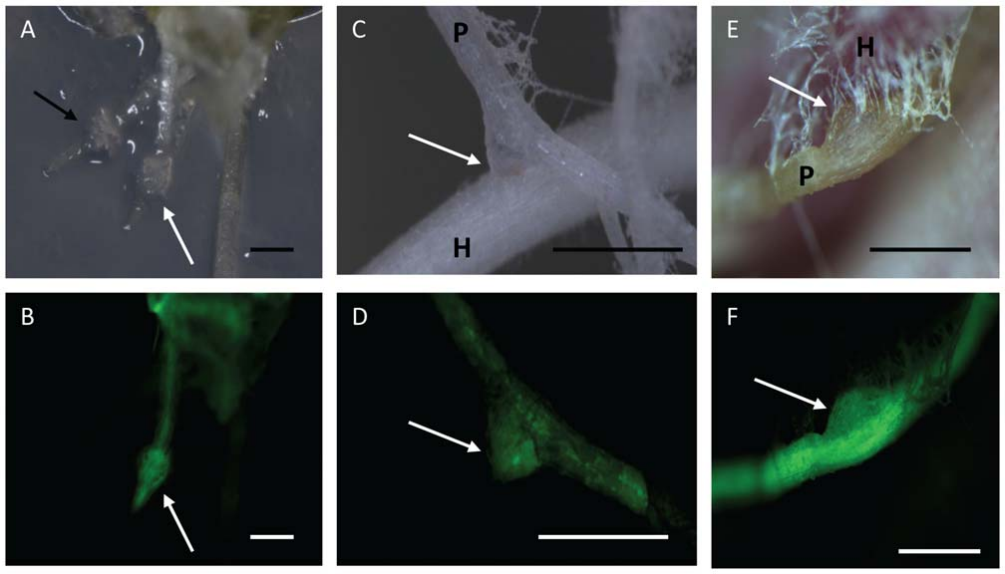
Transgenic root retains parasitic competence. **A** and **B**. Haustorium development in transgenic hairy roots following 2-day exposure to 10 µM DMBQ observed under bright field (A) and fluorescent (B) microscopy. The white arrows point to haustoria developing on a transformed root and the black arrow points to a haustorium in a non-transformed root. **C** and **D**. Haustorial connection with host rice observed under bright field (C) and fluorescence (D) microscopy. **E** and **F**. Haustorial connection with host maize observed under bright field (E) and fluorescence (F) microscopy. White arrows indicate haustorial connection of transgenic roots to hosts. H: host, P: parasite. Bars correspond to 0.5 mm.

To determine whether or not transgenic roots are able to form haustoria, transgenic roots were placed in a medium containing 10 µM DMBQ for 2 days. The region just proximal to the root tips swelled and became surrounded by root hair-like structures, which is typical for early haustorium development. Haustoria formed in transgenic roots were morphologically indistinguishable from those formed on non-transgenic roots (Fig. 6A, B). To test if transgenic *P. japonicum* roots can infest host plants, they were co-incubated with rice and maize *in vitro* or in non-aseptic conditions. In both cases haustoria developed and attached to the hosts apparently as well as wild type (Fig. 6C - F). The morphologically normal development of haustoria in *P. japonicum* hairy roots indicates that the hormonal effects caused by insertion of *rol* genes [28] do not influence the parasitic competence of the transformed roots.

#### Specific cell division during haustorial development

**Figure 7:**
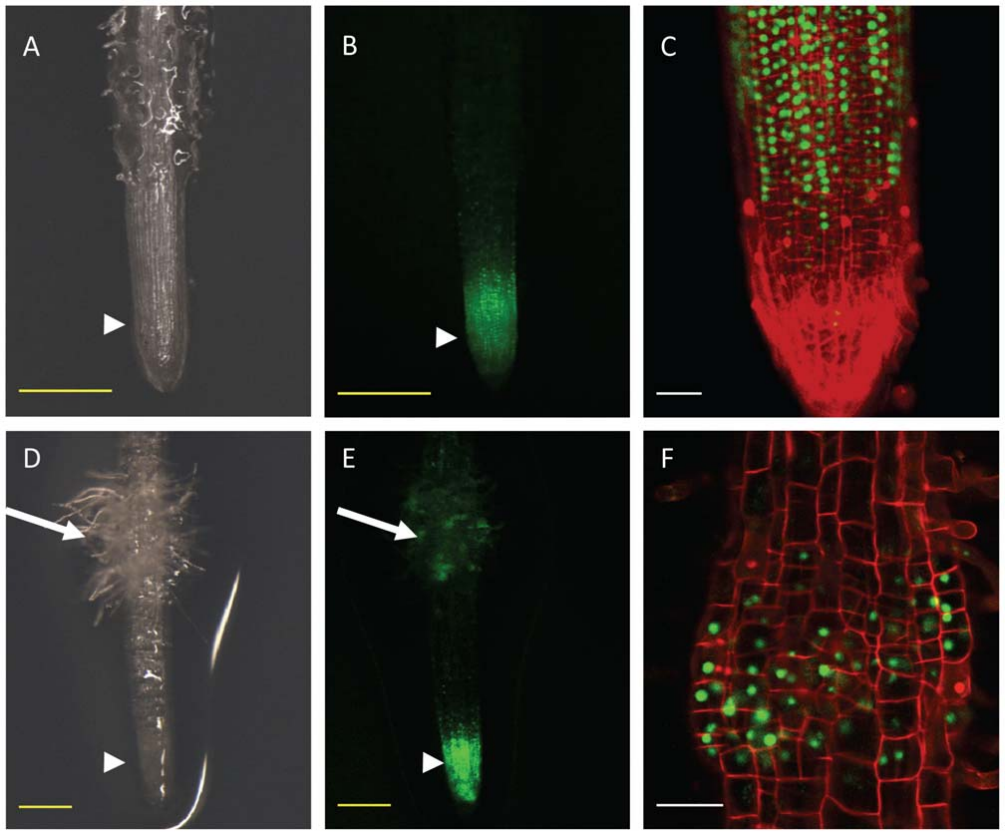
Cell division is induced during early haustorium development. Hairy root transformed with CYCB1;2 pro::YFP treated or not treated with 10 µM DMBQ. **A** to **C**. Transformed root without DMBQ treatment. YFP-fluorescing transgenic roots observed under bright field (A) or fluorescence (B) stereoscopy. In magnified view of root tip under fluorescence (C), roots were stained with 400 µg/ml propidium iodide (PI) to highlight root cell morphology. **D** to **F**. Transformed root after 24 h on DMBQ-containing agar (w/v 0.7%). Haustorium region in bright field (D) and under fluorescence (E) stereoscopy. PI-stained haustorium observed under fluorescence (F). The long arrows point to haustoria and the arrowheads to root tips. Red color indicates PI staining and green indicates GFP fluorescence. Yellow bar corresponds to 0.25 mm and white bar to 50 µm.

Using the established transformation protocol, we generated transgenic *P. japonicum* roots carrying the CYCB1;2 pro::YFPnuc construct that can be used to express a nucleus-localised yellow fluorescence reporter gene under the control of the *Arabidopsis Cyclin B1;2* promoter. The promoter region contains M-phase-specific *cis* elements widely-conserved in angiosperms as a general cell-division marker in heterologous systems [29]. We consistently observed intense fluorescence at the meristem, where cell divisions are normally active (Fig. 7 A and B). Confocal laser-scanning microscopy confirmed that fluorescence was restricted to the nuclei of meristematic cells (Fig. 7 C and D). Thus, our transformation system coupled with CYCB1;2 pro::YFPnuc is well-suited for visualizing cell division in *P. japonicum* roots.

To study cell division during haustorial development, *P japonicum* seedlings transformed with CYCB1;2 pro::YFPnuc were placed on DMBQ-containing agar. Twenty four hours after transfer to DMBQ agar, the region above the meristem was largely swollen (Fig. 7 E) and surrounded by root hair-like structures emitting strong fluorescence, indicating active cell divisions (Fig. 7 G and H). Our data showed that once the DMBQ signal from the host is perceived, particular cells initiate cell division and develop the haustorium.

### Conclusions

We have established an efficient *A. rhizogenes*-mediated transformation method for the facultative parasitic plant *P. japonicum* using sonication followed by vacuum treatment. This method has the advantage of rapid bacterial inoculation into plant tissue without the laborious need for excising hypocotyls or for needle inoculation. Transgenic roots can be obtained within a relatively short time (4–5 weeks) with an efficiency of around 20%. Furthermore, upon contact with a host or the HIF DMBQ, these roots develop haustoria morphologically indistinguishable from non-transformed ones. The transgenic haustoria apparently retain their ability to invade and parasitize host plants. Thus, our system provides a powerful genetic tool for investigating genes that function in the host-parasite interaction. In addition, we observed cell division during haustorium formation. Genes that function in these actively-dividing cells will be investigated in the near future using the system described in this report.

## Materials and Methods

### Bacterial strains

Bacterial strains used in this study are listed in Table 1. *Agrobacterium* strains were grown on LB media and cultured at 28°C for 2 days, except *A. rhizogenes* ATCC15384 which was cultured for 1 week. For infection, bacteria were suspended in MS salt solution supplemented with 2% (w/v) sucrose; 1x B5 vitamins; 0.02% (v/v) Silwet L-77, 100 µM acetosyringone (AS) and 0.5 mg/l NAA (pH 5.7), unless otherwise described, and the OD_600_ adjusted to 1.0.

#### Plant materials and growth conditions

*P. japonicum* (Thunb.) Kanitz seeds originally provided by Dr. T. Enomoto (Okayama University, Japan) [30] were self-crossed more than three times in laboratory. *P. japonicum* seeds were surface-sterilized with 5% (v/v) commercial bleach solution (approx. 6% sodium hypochlorite; Kao, Tokyo, Japan) with 0.1% (v/v) Triton X-100 (Wako Pure Chemical Industries, Ltd) for 5 minutes. After washing with excess water, seeds were placed on GM media, kept overnight in the dark at 4°C, and then transferred to 25°C for 3 days in darkness. After germination, plants were grown in a chamber with a photoperiod of 16 h light/8 h dark at 25°C. Commercial maize (*Zea mays*) seeds were sown directly on a moistened filter paper and incubated in a growth chamber at 26°C under a photoperiod of 16 h light/8 h dark. Rice seeds (*Oryza sativa*) were washed in 70% ethanol for 30 s, sterilized in 20% sodium hypochlorite solution for 15****min, rinsed with sterile water, and then incubated in moistened filter paper at 26°C under a photoperiod of 16 h.

#### Plasmids

The pBCR101 plasmid harbouring sGFP (S65T) gene driven by the CaMV35S promoter and terminated by a nopaline synthase polyadenylation signal was a kind gift of Drs. Seki and Muranaka (Osaka Univ). The construct was made in a pBI101 (Clontech) backbone by conventional cloning methods. For constructing CYCB1;2 pro::YFP, we used the pBGYN binary vector which contains the GATEWAY cassette (Invitrogen) fused to the 5′ end of YFP-NLS [31]. We first amplified the 5′ upstream region of CYCB1;2 by PCR using the primer set: 5′-CACCATCGTGAAGGTAACATTTACAAC-3′ and 5′-TTCTCTTTCGTAAAGAGTCTCTGCG-3′. The resulting 1.1-kb fragment containing the CYCB1;2 promoter and a part of adjacent gene was subcloned into a pENTR/D/TOPO vector (Invitrogen), and then integrated into pBGYN using LR clonase (Invitrogen).

#### *A. rhizogenes*-mediated transformation

*Injury-based methods* Five-day-old *P. japonicum* seedlings were transferred to Petri dishes with filter paper moistened with 2 ml of *A. rhizogenes* suspension (OD_600_ 1.0). The bacteria were inoculated into plant tissues by cutting the root off or injecting about 20 µl of the bacterial suspension directly into plant tissue using a hypodermic needle (26 GX 1/2” 0,45×13 mm – Terumo). The seedlings were transferred in B5 media and kept in the dark at 26°C for 3 days. After co-cultivation, the plants were placed in B5 media with cefotaxime (300 µg/ml).

*SAAT method*- Three-day-old *P. japonicum* seedlings were transferred to 15 ml plastic tubes containing 3–5 ml of *A. rhizogenes* suspension. Sonication treatment was carried out using a bath sonicator (Ultrasonic automatic washer, AS ONE, Japan) at room temperature for 10 s unless otherwise indicated. Seedlings were removed from the tubes and placed in Petri dishes containing filter paper moistened with bacterial suspension. The dishes were sealed with two turns of surgical tape and then submitted to a continuous vacuum for 5 minutes. The seedlings were transferred to co-cultivation media (B5 media, 1% (w/v) sucrose, 100 µM AS) and kept in the dark at 22°C for 7 days, unless other periods were described. After co-cultivation, plants were placed onto square Petri dishes containing B5 agar media supplemented with antibiotics cefotaxime (300 µg/ml). The dishes were sealed with surgical tape and incubated at 25°C vertically for 3 weeks with the bottom half being covered with aluminium foil. Transformation efficiency was calculated by counting cotyledon leaves that fluoresced under a stereomicroscope (Leica MZ16FA).

#### Genomic DNA extraction and PCR/RT-PCR amplification

Total genomic DNA was extracted from 100 mg *P. japonicum* hairy roots with a Phytopure DNA extraction kit (GE healthcare, Little Chalfont, England). The *GFP* fragment was amplified using the following primers: GFP Fwd 5′ – CTGACCCTGAAGTTCATCTGC-3′ and GFP Rev 5′- TCTTCTGCTTGTCGGCCATG-3′. Amplification was performed with an initial hot start at 94°C for 3 min, then 35 cycles of denaturation (94°C 15 s), annealing (55°C 15 s), extension (72°C 30 s), and a final extension of 5 min at 72°C. Ten pg of pBCR101 plasmid was used as the positive control. The sequences of *virD1* and *rolB*-specific primers and the amplification protocols have been described [32]. Briefly, the *rolB* primers amplified a 780 bp fragment of the T*_L_* region from the Ri plasmid of *A. rhizogenes*, and the *virD1* primers amplified a fragment size of 450 bp. The PCR amplification products were analyzed by electrophoresis in 1% agarose gels containing ethidium bromide. For a positive control, a diluted *A. rhizogenes* (ATCC15834 containing pBCR101) suspension was used.

#### Southern blot analysis

The genomic DNA from transformed or non-transformed *P. japonicum* tissue was extracted with a Phytopure DNA extraction kit (GE healthcare, Little Chalfont, England) according to the manufacturer's instructions. Sixteen µg of the genomic DNA was digested with *Eco*RI. The 380 bp GFP fragment was amplified using the GFP Fwd and GFP Rev primer pairs as described above and labelled using an AlkPhos direct kit (GE healthcare). Pre-hybridization, hybridization and washing were performed according to the manufacturer's instructions. For a positive control pBCR 101 containing the *GFP* gene was digested with *Xho*I and 30 ng of linear plasmid was loaded onto the gel.

#### Haustorium assays

Haustorium development was monitored in *P. japonicum* transgenic roots after contact with rice and maize roots. Rice seeds with their seed coats removed were immersed in 70% ethanol for 1 min and then in a 20% commercial hypochlorite solution for 20 min with agitation, followed by washing five times with sterile water. The rice seeds were then germinated on moistened filter paper for 1 week, transferred to 0.7% (w/v) agar in a square plate, positioned vertically and grown for 1 week at 25°C in a 16-h light/8-h dark photo period. Transformed *P. japonicum* roots were placed close to the host roots, and haustorium development was monitored. One week after maize germination on moistened filter paper, *P. japonicum* plants with transgenic roots were placed in the rhizotron system as described previously [30]. For DMBQ treatment, *P. japonicum* roots were transferred to 0.7% (w/v) agar containing 10 µM DMBQ.

#### Microscopy

GFP and YFP fluorescence in roots was observed under a Leica MZ16FA stereomicroscope. Cotyledon surfaces were visualized *in natura* with a scanning electron microscope (Miniscope TM1000, Hitach High-Technologies, Japan). In the confocal laser-scanning microscopy analysis, *P. japonicum* seedlings carrying the CYCB1;2 pro::YFP construct were treated with or without DMBQ as described above. The transformed roots were stained with 5 µM propidium iodide solution (Wako Chemical, Japan) and immediately observed with a Zeiss LSM510 Meta microscope.
